# The enzymes LSD1 and Set1A cooperate with the viral protein HBx to establish an active hepatitis B viral chromatin state

**DOI:** 10.1038/srep25901

**Published:** 2016-05-13

**Authors:** Valentina Alarcon, Sergio Hernández, Lorena Rubio, Francisca Alvarez, Yvo Flores, Manuel Varas-Godoy, Giancarlo V. De Ferrari, Michael Kann, Rodrigo A. Villanueva, Alejandra Loyola

**Affiliations:** 1 Fundación Ciencia & Vida, 7780272, Santiago, Chile; 2 Laboratorio de Virus Hepatitis, Departamento de Ciencias Biológicas, Facultad de Ciencias Biológicas, Universidad Andres Bello, Santiago, Chile; 3 Centro de Investigación Biomédica, Facultad de Medicina, Universidad de los Andes, Santiago, Chile; 4 Centro de Investigaciones Biomédicas. Facultad de Ciencias Biológicas y Facultad de Medicina. Universidad Andres Bello, Santiago, Chile; 5 Univ. de Bordeaux, Microbiologie Fondamentale et Pathogénicité, UMR 5234, Bordeaux, France; 6 CNRS, Microbiologie Fondamentale et Pathogénicité, UMR 5234, Bordeaux, France; 7 CHU de Bordeaux, Bordeaux, France; 8 Universidad San Sebastián, Santiago, Chile

## Abstract

With about 350 million people chronically infected around the world hepatitis B is a major health problem. Template for progeny HBV synthesis is the viral genome, organized as a minichromosome (cccDNA) inside the hepatocyte nucleus. How viral cccDNA gene expression is regulated by its chromatin structure; more importantly, how the modulation of this structure impacts on viral gene expression remains elusive. Here, we found that the enzyme SetDB1 contributes to setting up a repressed cccDNA chromatin state. This repressive state is activated by the histone lysine demethylase-1 (LSD1). Consistently, inhibiting or reducing LSD1 levels led to repression of viral gene expression. This correlates with the transcriptionally repressive mark H3K9 methylation and reduction on the activating marks H3 acetylation and H3K4 methylation on viral promoters. Investigating the importance of viral proteins we found that LSD1 recruitment to viral promoters was dependent on the viral transactivator protein HBx. Moreover, the histone methyltransferase Set1A and HBx are simultaneously bound to the core promoter, and Set1A expression correlates with cccDNA H3K4 methylation. Our results shed light on the mechanisms of HBV regulation mediated by the cccDNA chromatin structure, offering new therapeutic targets to develop drugs for the treatment of chronically infected HBV patients.

Hepatitis B virus (HBV) infection remains a major health problem worldwide, in spite of the existence of an effective vaccine since the eighties. The World Health Organization estimates that more than 350 million people worldwide suffer from chronic hepatitis caused by HBV infection^1^,^2^. In these patients, the virus replicates persistently, which can result in cirrhosis and hepatocellular carcinoma (HCC). Although there is a correlation between HBV infection and the development of HCC, the molecular mechanisms involved remain elusive^3^.

HBV is an enveloped pararetrovirus containing a partially double stranded DNA genome (relaxed circular DNA, rcDNA), which is surrounded by the viral capsid. The infective cycle starts with the binding of HBV to its cellular receptor, the sodium taurocholate cotransporting polypeptide (NTCP) protein, localized in the hepatocyte^4^. The virus is incorporated into the cell using endocytosis followed by release of the capsid and its transport to the nucleus. After passing the nuclear pores the capsid dissociates leading to genome release. The genome is repaired to a covalently closed circular double-strand DNA (cccDNA) of 3.2 kb. This cccDNA is the replicative intermediate serving as a template for the transcription of all the viral transcripts, including the pregenomic RNA^5^. The cccDNA molecule, responsible for persistent infection, is kept in the nucleus of the infected hepatocyte as an episomal DNA^6^. It is organized as a minichromosome by its association with cellular histones and non-histone proteins^7^,^8^. Recent reports revealed that the cccDNA chromatin structure regulates the HBV replication and transcription^9^,^10^,^11^,^12^,^13^,^14^,^15^, utilizing host mechanisms that control cellular genome expression^16^,^17^. Indeed, acetylation of histones H3 and H4 bound to the cccDNA plays a critical role in HBV expression. There is a temporal correlation between acetylation and the level of HBV replicative intermediates^9^. Moreover, in chronically infected patients, the acetylation status of histones H3 and H4 correlates with viremia levels^9^. Furthermore, IFN-α, clinically used as an inhibitor of cccDNA transcription and HBV replication, leads to hypoacetylation of histones bound to the HBV cccDNA^10^,^11^.

The viral protein HBx plays a pivotal role in HBV viral transcription. On one hand it regulates the degradation of the structural maintenance of chromosomes (SMC) complex Smc5/6 avoiding its binding and repression of viral cccDNA^18^. On the other hand it modulates the cccDNA chromatin state by regulating the recruitment of chromatin modifying enzymes^11^,^14^,^19^,^20^. In its absence, the viral genome exists in a repressed chromatin state. The histone deacetylases HDAC1 and hSirt1, but not histone acetyltransferase p300, are bound to viral promoters on HBV X(-) mutant virus^11^, a virus that is impaired in viral replication^21^. Consistently, cccDNA bound histones H3 and H4 are hypoacetylated in the HBV X(-) mutant^11^. In addition to histone hypoacetylation, the silencing mechanism involves methylation of histone H3 on lysine 9 (H3K9me), a hallmark of heterochromatin, by the methyltransferase SetDB1; and the recruitment of the heterochromatin protein HP1^20^. In the presence of viral protein HBx the cccDNA forms an active chromatin state, with reduced levels of H3K9me^20^. However, the mechanisms involved in this chromatin state switching process remain elusive. Thus, we focused on histone demethylase lysine-specific demethylase-1 (LSD1). LSD1 protein can mediate either transcriptional repression or activation by demethylating H3K4me1/2 or H3K9me1/2, respectively^22^. Indeed, reactivation of the herpes virus from latency involves the recruitment of LSD1, which demethylates the repressive mark H3K9me2 present on the repressed immediate early herpes viral promoter genes, allowing transcriptional activation^23^,^24^. In this report, we utilized an *in vitro* HBV replication model system that uses a HBV genotype F clone^15^,^25^ to investigate whether LSD1 activates HBV transcription. Interestingly, we found that viral protein HBx recruits LSD1 to HBV viral promoters correlating with a reduction of the methylation on H3K9. In addition, HBx recruits the enzyme Set1A to viral promoters to trimethylate H3K4, a mark associated with transcriptional activation, thus helping to establish an active viral chromatin state.

## Results

### SetDB1 represses HBV transcription

**Figure 1:**
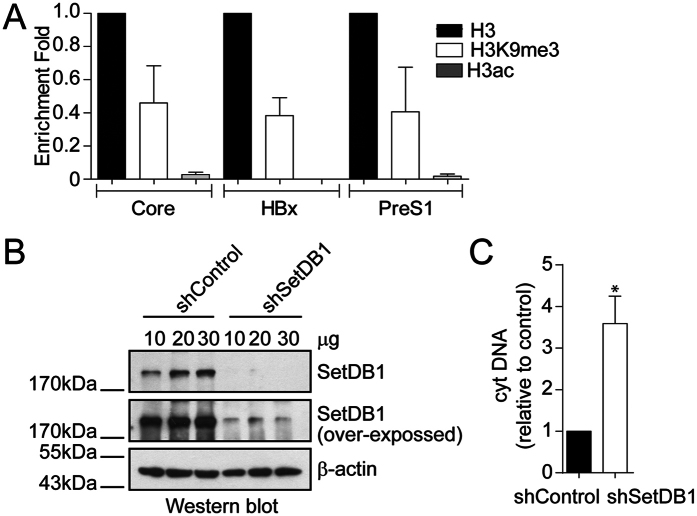
SetDB1 represses HBV transcription. (**A**) Covalent post-translational modifications on histone H3 were determined by Chromatin Immunoprecipitation (ChIP) analysis using specific antibodies, as indicated. Immunoprecipitated DNA was quantified by qPCR using specific primers as detailed in Material and Methods. The results are expressed as enrichment fold change with respect to H3. The standard deviation was obtained from three independent experiments. (**B**) SetDB1 knock down was examined by Western blot using specific antibodies. Beta actin was used as a loading control. (**C**) HBV cytoplasmic intermediate levels were determined by qPCR using specific primers. The result is shown as fold changes with respect to the control. The standard deviation was obtained from three independent experiments. *p < 0.05, Student´s *t*-test.

We investigated the chromatin state of the HBV genome in an *in vitro* HBV replication model system based on the transfection of linear HBV genome monomeric molecules containing cohesive ends that allows its circularization^15^. We performed a Chromatin Immunoprecipitation (ChIP) assay of two covalent post-translational modifications, H3K9me3 and H3ac, associated with transcriptional repression and activation, respectively. We then performed quantitative PCR to examine the presence of these marks on histones bound to three HBV promoters: the proximal promoter region of the core viral gene, which drives transcription of the pre-genomic RNA; the HBx promoter, which gives rise to the viral regulatory protein HBx, and the preS1 promoter, which drives transcription of the LHBsAg^5^. We found that the H3K9me3 mark was enriched on the three viral promoters, whereas H3ac was low or undetected (Fig. 1A). This result suggests that the HBV genome is organized in a repressed chromatin state. We then investigated whether the enzyme SetDB1 was involved in repressing HBV transcription, as recently shown^20^. Thus, we specifically reduced the levels of SetDB1 protein by using shRNA to knock down SetDB1 mRNAs (shSetDB1). We performed the experiment by treating the cells with and without shSetDB1 48 h prior to transfecting the HBV genome. Western blot analysis confirmed a reduction in SetDB1 protein levels after the treatment (Fig. 1B). We then examined the effect of shSetDB1 on HBV transcription by qPCR using specific primers. For this, we analyzed cytoplasmic viral core particles and found that, upon shSetDB1 treatment, viral particles DNA increased significantly (Fig. 1C). Therefore, the data indicate that SetDB1 negatively regulates the transcription of viral promoters.

### LSD1 increases the levels of HBV replicative intermediates

**Figure 2:**
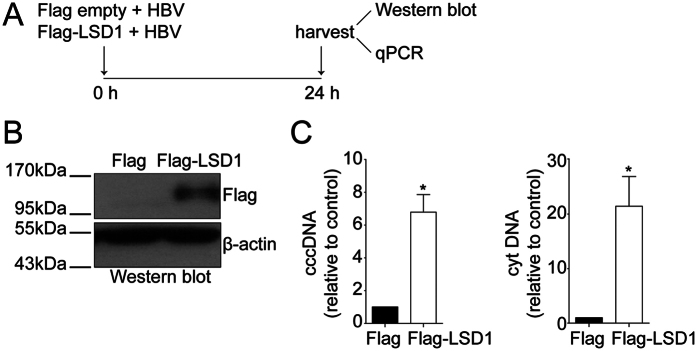
LSD1 increases the levels of HBV replicative intermediates. (**A**) The scheme depicts the experimental procedure to overexpress LSD1. Human hepatocarcinoma Huh7 cells were transfected with the HBV genome together with either a plasmid encoding a Flag-tagged LSD1 or a Flag protein as a control. (**B**) Western blot analysis to monitor LSD1 overexpression. Beta actin was used as a loading control. (**C**) HBV cccDNA and cytoplasmic intermediate levels were determined by qPCR using specific primers. The results are shown as fold changes with respect to the control. The standard deviation was obtained from three independent experiments. *p < 0.05, Student´s *t*-test.

We then explored the molecular mechanisms by which repressed viral promoters become active. Given that the herpes virus regulates its reactivation from latency in part by recruiting LSD1 to demethylate H3K9me2 on repressed immediate early herpes viral promoter genes^23^,^24^, and that histone H3 bound to the HBV cccDNA is methylated at residue K9 (Fig. 1A), we investigated the effect of LSD1 on HBV replication. For this, we co-transfected Huh7 cells with the plasmid-free HBV genome plus either a plasmid encoding Flag-tagged LSD1 or the empty vector as a negative control, as illustrated in Fig. 2A. Western blot analysis confirmed that after 24 h LSD1 was overexpressed in the cells (Fig. 2B). We then isolated the HBV replicative intermediates cccDNA and cytoplasmic viral core particles from transfected cells and quantified the viral DNA intermediates. We observed that the levels of both viral intermediates increased significantly upon overexpressing LSD1 (Fig. 2C). Thus, LSD1 increases the levels of HBV replicative intermediates.

### Inhibition of LSD1 represses HBV transcription and replication

**Figure 3:**
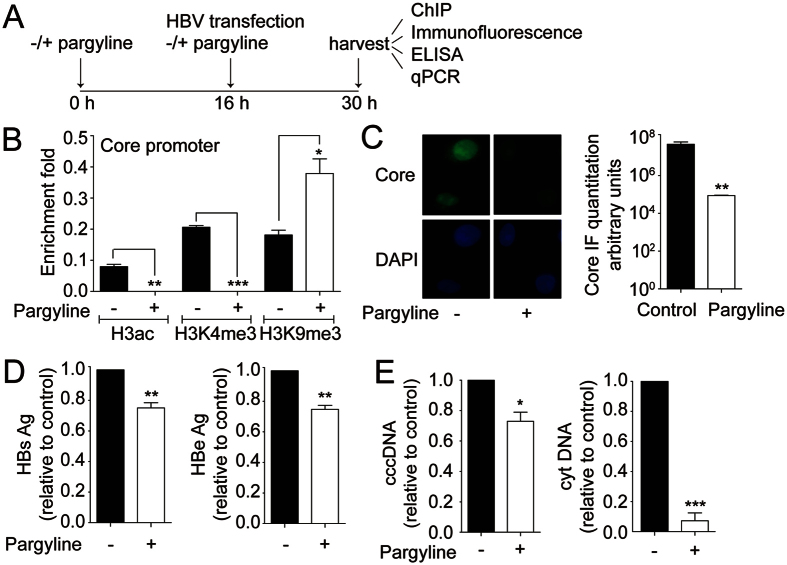
Inhibition of LSD1 represses HBV transcription and replication. (**A**) The scheme depicts the experimental procedure. Human hepatocarcinoma Huh7 cells were grown in the presence or absence of 0.5 mM of the LSD1 inhibitor pargyline. 16 h after, cells were transfected with the HBV genome in the presence or absence of pargyline. (**B**) Covalent post-translational modifications on histone H3 were determined by Chromatin Immunoprecipitation (ChIP) analysis using specific antibodies as indicated. Immunoprecipitated DNA was quantified by qPCR using specific primers to the core promoter as detailed in Material and Methods. Histone modifications were normalized against the immunoprecipitated H3. The standard deviation was obtained from three PCR reactions and the graph is representative of three independent experiments. (**C**) Immunofluorescence analyses of pargyline treated and untreated cells were performed using specific antibodies against the core protein and DAPI staining. On the right, the graph shows pixel intensity in arbitrary units. The staining intensity was quantified on at least 100 cells using Image J software. Standard deviation was obtained from three independent experiments. (**D**) The quantity of viral antigens in the supernatant of the transfected cells was determined by ELISA, using specific antibodies against the HBs and HBe proteins. The results are shown as fold changes with respect to the control. The standard deviation was obtained from three independent experiments. (**E**) HBV cccDNA and cytoplasmic intermediate levels were determined by qPCR using specific primers. The results are shown as fold changes with respect to the control. The standard deviation was obtained from three independent experiments. *p < 0.05, **p < 0.01, ***p < 0.001, Student´s *t*-test.

To investigate further whether LSD1 regulates the transcription and replication of HBV, we utilized an LSD1 inhibitor. LSD1 demethylates lysine residues via a Flavin-adenine dinucleotide-dependent reaction that is inhibited by monoamino oxidase inhibitors (MAOI) such as pargyline^23^. Thus, Huh7 cells were transfected with the HBV genome in the presence or absence of 0.5 mM pargyline, as illustrated in Fig. 3A. Pargyline did not affect cell viability, as determined by trypan blue staining (Supplementary Figure 1A). We decided to analyze by ChIP covalent post-translational modifications of the histones located on the proximal promoter region of the core viral gene, because it plays an important role in the regulation of HBV replication. Interestingly, after 24 h treatment, the histone modifications on the core promoter switched. Histone H3 became hypoacetylated and displayed lower levels of H3K4me3 compared to untreated cells. In contrast, the levels of H3K9me3 increased (Fig. 3B). Thus, ChIP analyses indicate that pargyline changed the state of the core viral gene promoter, switching from an activated to a repressed state. We then examined the effect of pargyline on the viral protein expression. We performed immunofluorescence assays and observed reduction of capsids upon pargyline treatment, consistent with the ChIP results (Fig. 3C). Furthermore, the examination of the HBs and HBe viral antigens, used as markers of acute infection and active replication, respectively, showed that pargyline gave rise to a reduction of both viral antigens levels (Fig. 3D). We finally investigated the effect of pargyline on HBV replicative intermediates and found that cccDNA and cytoplasmic viral core particles significantly decreased (Fig. 3E). Taken together, the data suggest that LSD1 regulates the HBV cycle, through a mechanism that involves the demethylation of H3K9, switching the cccDNA chromatin state from a repressed to an activated state.

### Knock down of LSD1 represses HBV transcription and replication

**Figure 4:**
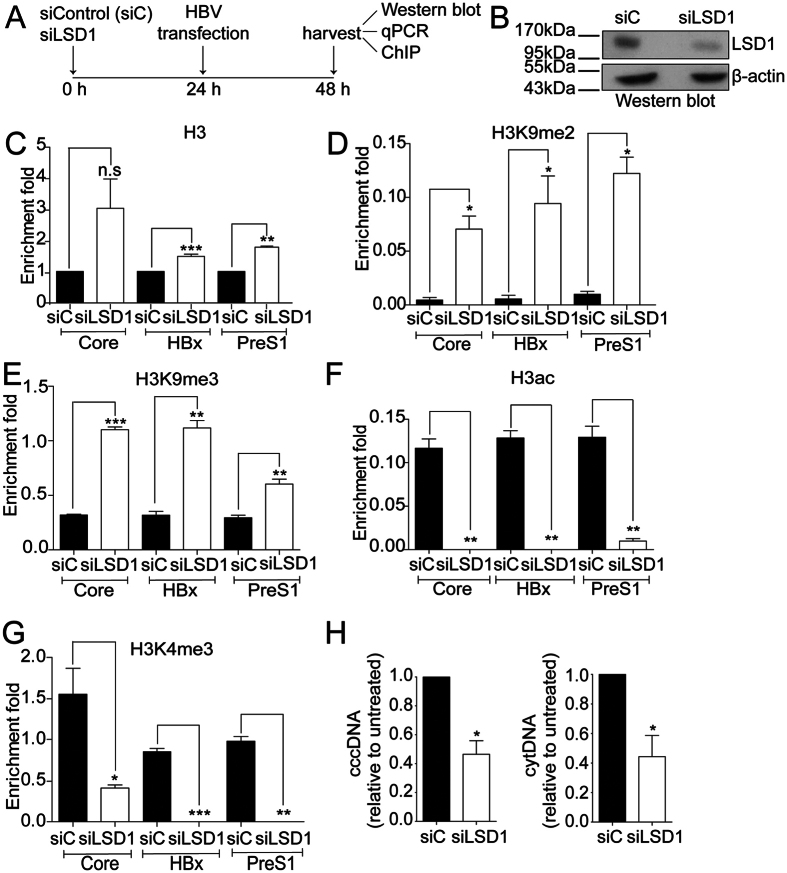
Knock down of LSD1 represses HBV transcription and replication. (**A**) The scheme depicts the experimental procedure. Human hepatocarcinoma Huh7 cells were transfected with 10 nM of either control or LSD1 siRNA. 24 h after, cells were transfected with the HBV genome. (**B**) LSD1 knock down was determined by Western blot using specific antibodies. Beta actin was used as a loading control. (**C–G**) Covalent post-translational modifications on histone H3 were determined by ChIP analysis using the specific antibodies as indicated. Immunoprecipitated DNA was quantified by qPCR using specific primers as detailed in Material and Methods. When analyzing histone modifications, we normalized the data against the immunoprecipitated H3. The standard deviation was obtained from three PCR reactions and the graphs are representative of three independent experiments. (**H**) HBV cccDNA and cytoplasmic intermediate levels were determined by qPCR using specific primers. The results are shown as fold changes with respect to the control. The standard deviation was obtained from three independent experiments. *p < 0.05, **p < 0.01, ***p < 0.001, Student´s *t*-test.

To demonstrate that the effects observed with pargyline were due to LSD1 inhibition, we specifically reduced the levels of LSD1 protein by using siRNA (siLSD1). We performed the experiment as illustrated in Fig. 4A, by treating the cells with and without siLSD1 24 h prior to transfecting the HBV genome. Western blot analysis confirmed the LSD1 reduction after the treatment (Fig. 4B). ChIP assay showed that histone H3 levels were enriched in two viral promoters upon LSD1 reduction (Fig. 4C). We then examined histone post-translational modifications. Because the levels of histone H3 varied between untreated and siLSD1 treated cells, we normalized the H3 modification ChIP results against the H3 value for each sample, as explained in Material and Methods. We observed that H3K9 di- and trimethylation (H3K9me2 and H3K9me3) were significantly enriched on the three promoters (Fig. 4D,E). Consistent with the enrichment of repressive marks, H3ac and H3K4me3 modifications that correlate with transcriptional activation were decreased (Fig. 4F,G). We then examined whether the changes in histone modifications on viral promoters mediated by siLSD1 impacted on HBV transcription and replication. For this, we analyzed HBV replicative intermediates cccDNA and cytoplasmic viral core particles and found that both intermediates decreased significantly (Fig. 4H). Taken together, the data indicate that LSD1 positively regulates transcription of viral promoters by activating the cccDNA chromatin state.

### LSD1 is recruited to HBV promoters in an HBx-dependent manner

**Figure 5:**
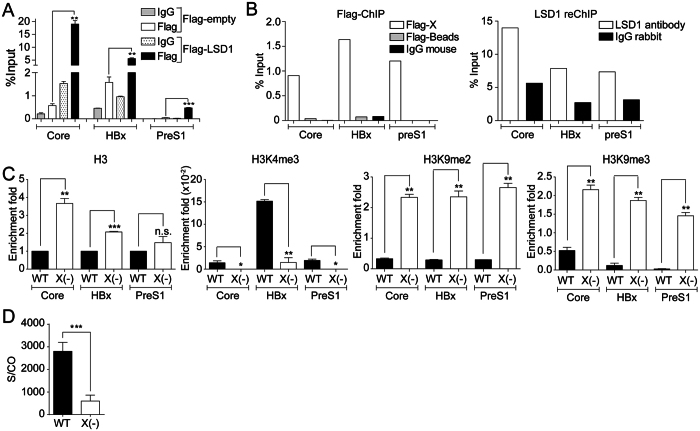
LSD1 is recruited to HBV promoters in an HBx-dependent manner. (**A**) Human hepatocarcinoma HepG2 cells were transfected with the wild type HBV genome together with either a plasmid encoding for a Flag-tagged LSD1 or a Flag protein as a control. Flag-LSD1 recruitment to viral promoters was assayed by ChIP analysis. Immunoprecipitated DNA was quantified by qPCR using specific primers as detailed in Material and Methods. The results are expressed as % of input. The standard deviation was obtained from three independent experiments. (**B**) Human hepatocarcinoma HepG2 cells were transfected with the wild type HBV genome together with either a plasmid encoding for a Flag-tagged HBx or a Flag protein as a control. Flag-HBx recruitment to viral promoters was assayed by ChIP analysis (left). Then the Flag-HBx immunoprecipitated samples were reChIP with antibodies against LSD1 or IgG antibodies as control (right). Immunoprecipitated DNA was quantified by qPCR using specific primers as detailed in Material and Methods. The results are expressed as % of input. (**C**) Covalent post-translational modifications on histone H3 were determined by ChIP analysis using the specific antibodies, as indicated. Immunoprecipitated DNA was quantified by qPCR using specific primers as detailed in Material and Methods. When analyzing histone modifications, we normalized the data against the immunoprecipitated H3. The standard deviation was obtained from three PCR reactions and the graphs are representative of three independent experiments. (**D**) The quantity of viral antigens in the supernatant of WT and HBV- transfected cells after 72 h was determined by ELISA, using specific antibodies against the HBs protein. In each case, the results are presented as a signal-to-cutoff (S/CO) ratio. The standard deviation was obtained from four independent experiments. *p < 0.05, **p < 0.01, ***p < 0.001, Student´s *t*-test.

To investigate how LSD1 activates viral transcription, we first explored whether LSD1 binds to viral promoters. We overexpressed Flag-tagged LSD1 and performed Flag-ChIP analysis. We found a specific binding of LSD1 on the three viral promoters studied (Fig. 5A and Supplementary Figure 1B). LSD1 cannot directly interact with DNA and it is recruited to promoter regions by interacting with transcriptional regulators^26^. Given that the viral HBx protein regulates the binding of several chromatin modifying enzymes to the cccDNA^11^,^14^,^19^, we investigated whether HBx and LSD1 bind to the promoters at the same time by reChIP. We co-transfected the HBV genome with a vector expressing either a tagged version of HBx (Flag-HBx) or an empty vector. We then performed Flag-ChIP assays and found Flag-HBx on the three viral promoters (Fig. 5B, left). We then immunoprecipitated the Flag-HBx ChIP samples with an antibody against LSD1 (Fig. 5B, right) and found LSD1 present in promoters containing HBx protein. We conclude that HBx and LSD1 bind to viral promoters at the same time. To confirm the role of HBx, we took advantage of a mutant HBV genome carrying a stop codon in amino acid 8 of the HBx open reading frame (HBV X-)^11^ and introduced the mutation on the HBV genotype F genome. We performed ChIP assays in the absence of HBx. Consistent with a previous report^20^, viral promoters were enriched on histone H3 as well as on the marks H3K9me2 and H3K9me3, in the absence of HBx (Fig. 5C). In contrast, the viral promoters showed reduced levels of H3K4me3 in the absence of HBx (Fig. 5C). As expected, the HBV X- cccDNA chromatin state correlated with a repressed HBV gene expression, as illustrated with HBs viral antigen levels (Fig. 5D and ref. 15). Thus, these results suggest that in the absence of HBx protein LSD1 is not recruited to cccDNA, leading to enrichment in H3K9 methylation and the establishment of a repressed HBV cccDNA chromatin state.

### Set1A participates in the establishment of the mark H3K4me3 on HBV promoters

**Figure 6:**
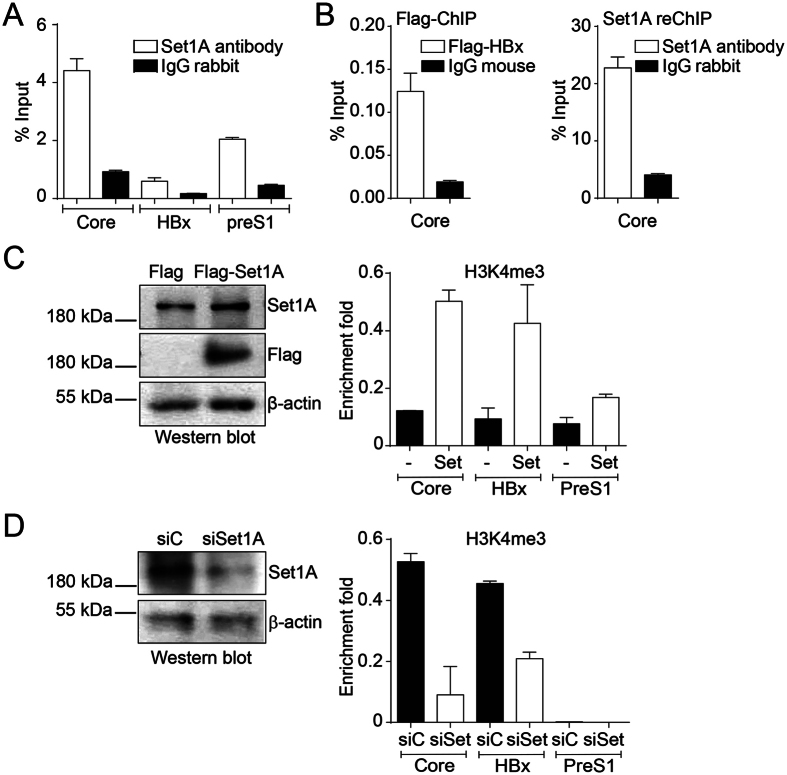
Set1A participates in the establishment of the mark H3K4me3 on HBV promoters. (**A**) Human hepatocarcinoma Huh7 cells were transfected with the wild type HBV genome. Set1A recruitment to viral promoters was assayed by ChIP analysis. Immunoprecipitated DNA was quantified by qPCR using specific primers as detailed in Material and Methods. The results are expressed as % of input. The standard deviation was obtained from two PCR reactions and the graph is representative of three independent experiments. (**B**) Human hepatocarcinoma HepG2 cells were transfected with the wild type HBV genome together with a plasmid encoding for Flag-tagged HBx. Flag-HBx recruitment to viral promoters was assayed by ChIP analysis (left). Then the Flag-HBx immunoprecipitated samples were reChIP with antibodies against Set1A or IgG antibodies as control (right). Immunoprecipitated DNA was quantified by qPCR using specific primers for core promoter. The results are expressed as % of input. (**C,D**, left) Western blot analyses to monitor overexpression (**C**) and knock down (**D**) of Set1A. Βeta actin was used as a loading control. (**C,D**) H3K4me3 modification was determined by ChIP analysis using specific antibodies from cells overexpressing (**C**) or knocking down (**D**) Set1A. Immunoprecipitated DNA was quantified by qPCR using specific primers as detailed in Material and Methods. We normalized the data against the immunoprecipitated H3. The standard deviation was obtained from two PCR reactions and the graphs are representative of three independent experiments.

Given that the active cccDNA chromatin state correlates with enrichment of the mark H3K4me3, whereas the repressed state with low levels of H3K4me3, we investigated how this modification is imposed on the HBV cccDNA. Thus, we first examined whether the H3K4 methyltransferase Set1A, the catalytic subunit of the H3K4 methyltransferase complex COMPASS^27^, binds to viral promoters. We performed ChIP analysis with antibodies against Set1A and found it specifically bound to the three viral promoters studied (Fig. 6A). We then investigated whether HBx and Set1A bind to the promoters at the same time by reChIP. We co-transfected the HBV genome and a vector expressing a tagged version of HBx (Flag-HBx). We then performed Flag-ChIP assays to isolate Flag-HBx bound cccDNA DNA (Fig. 6B, left), followed by Set1A-ChIP (Fig. 6B, right) and found Set1A present at core promoters containing HBx protein. We conclude that HBx and Set1A bind to viral promoters at the same time. We then investigated the effect of modulating Set1A cellular levels on cccDNA H3K4 methylation by overexpressing (Fig. 6C, left) or reducing (Fig. 6D, left) Set1A protein. ChIP assays showed that H3K4me3 was enriched on the three viral promoters upon Set1A overexpression (Fig. 6C, right). In contrast, knocking down Set1A resulted in the reduction of the mark H3K4me3 on viral promoters (Fig. 6D, right). Taken together, the results show that Set1A establishes the mark H3K4me3 on HBV promoters and that this mark can be altered by modulating Set1A levels.

## Discussion

In chronically infected patients the HBV genome remains in the nucleus of the hepatocyte as a minichromosome, structured similarly to the cellular chromatin. Despite the knowledge that has been gained about the impact of chromatin structure on gene expression, only a few reports have investigated how histone modification changes on the cccDNA minichromosome influence HBV gene expression^11^,^14^,^15^,^19^,^20^. In this study, we have shown that the enzyme SetDB1 contributes to establishing a repressed HBV cccDNA chromatin state, consistent with a recent report^20^. This repressed cccDNA chromatin state is activated by the enzymes LSD1 and Set1A. Our results suggest that LSD1 mediates its activation by demethylating H3K9, whereas Set1A by methylating H3K4. In addition, we showed that both LSD1 and Set1A are recruited to viral promoters in an HBx dependent manner.

**Figure 7:**
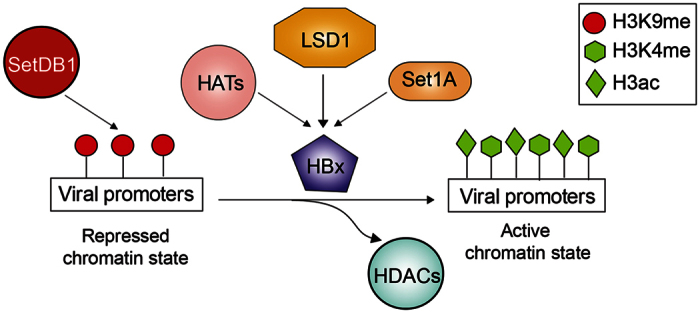
HBx contributes to establish an active cccDNA chromatin state. The viral HBx protein plays a key role in the establishment of an active cccDNA chromatin state by regulating the recruitment of chromatin modifying enzymes, including LSD1 and Set1A, as demonstrated here.

We found that 24 h after HBV genome transfection, histones H3 bound to HBV promoters are hypoacetylated and methylated at residue K9, both marks associated with transcriptional repression (Fig. 1). It has been suggested that the establishment of these marks is part of a cellular mechanism aimed at silencing the virus^20^. Therefore, in order for active HBV transcription and replication to occur, the repressed modifications need to be removed and active marks imposed on the HBV minichromosome. The viral HBx protein plays a key role in this activating mechanism by recruiting chromatin modifying enzymes, such as the histone acetyltransferase p300^11^,^14^,^19^,^20^. In its absence, histone deacetylases are recruited to the cccDNA instead^11^. Our results indicate that HBx is necessary for the recruitment of the enzymes LSD1 and Set1A to participate in the activation of the repressed HBV cccDNA chromatin state. Intriguingly, LSD1 can demethylate histone H3 on lysines 4 and 9, depending on the proteins that it interacts with. When LSD1 associates with the Co-REST complex it contributes to the establishment of transcriptional repression by removing H3K4 methylation. In contrast, when LSD1 associates with the androgen receptor, it establishes a transcriptionally active state by removing H3K9 methylation^28^. We showed that LSD1 is required for a reduction of the methylation levels of H3K9, but not H3K4, bound to the HBV cccDNA minichromosome; however, how LSD1 activity is regulated in the HBV context remains unknown. Further experiments should investigate whether androgen receptor and the HBx protein work together to regulate the association of LSD1 to viral promoters and its activity. Our results support the model proposed by the Levrero’s group^11^, in which the viral HBx protein regulates the cccDNA chromatin state by recruiting chromatin modifiers to viral promoters (Fig. 7), including the histone acetyltransferase p300, the histone demethylase LSD1, and the histone methyltransferase Set1A, as suggested in here. As a consequence, an active HBV cccDNA chromatin state is established that promotes HBV expression and production of viral progeny.

For the treatment of chronically infected patients there are currently two types of antivirals: interferon-α, which stimulates the immune system and can eventually clear HBV^29^, and nucleotide/nucleoside analogs, which interfere with the viral replication by inhibiting the viral polymerase/reverse-transcriptase activity. However, only 20–30% of the treated patients eradicate the infection, due to the persistence of the cccDNA in infected hepatocytes^30^. In addition, HBV has developed resistance to these type of drugs^31^. Therefore, it is necessary to develop new therapeutic targets and compounds. We and others have shown that the cccDNA minichromosome structure is dynamic and viral gene expression adapts its outcome according to changes on the chromatin including histone modifications. For instance, upon treatment with deacetylase inhibitors, the histones bound to the cccDNA become hyperacetylated, correlating with activation of viral gene expression^9^,^9^. Our results indicate that modulating the cccDNA chromatin structure towards a repressed chromatin state is a potential therapeutic approach for chronically infected HBV patients. We have shown that by inhibiting LSD1 demethylase with pargyline, an irreversible non-selective monoamine oxidase inhibitor, viral gene expression is repressed. Interestingly, pargyline is used in the clinic by patients suffering from hypertension; thus, it would be interestingly to explore its effect in an *in vivo* HBV animal model as a potential drug for chronically infected HBV patients. However, the use of pargyline in HBV chronically infected patients has the limitation that it is not a specific LSD1 inhibitor. Giving that LSD1 is overexpressed in many types of cancers and it is believed that its overexpression contributes to carcinogenesis, screening for LSD1 inhibitors has been intently pursued, thus specific LSD1 inhibitors have been developed^32^.

However, it should be pointed out that therapies based on the inhibition of cellular enzymes, such as LSD1, will most likely have side effects. Therefore, ways of blocking the recruitment of chromatin modifying enzymes, such as LSD1, specifically to the cccDNA minichromosome rather than inhibiting the enzyme would be a safer approach for patient treatment. Given the evidence that the viral protein HBx is required for the recruitment of many chromatin modifying enzymes to the cccDNA^11^,^14^,^19^, HBx protein would be a target to explore. Thus, our results highlight the importance of understanding how the cccDNA minichromosome is structured and how this structure as well as changes in it can control HBV gene expression and replication.

## Materials and Methods

### Antibodies, drugs, and primers

Antibodies: β-actin (Millipore #04-1116), FLAG (Sigma Aldrich #F3165-2MG), hepatitis B virus core antigen (Dako # B0586), histone H3 (Abcam #ab1791), H3ac (Merck #06-599), H3K4me3 (Abcam #ab8580), H3K9me2 (Abcam #ab1220), H3K9me3 (Abcam #ab8898), LSD1 (Abcam #ab17721), anti-FLAG beads (Sigma Aldrich #A2220), Set1A (Bethyl #A300-290A), SetDB1 (Abcam #ab12317). Drugs: Pargyline hydrochloride (sc-215676A, Santa Cruz Biotechnology). Primer cccDNA: Forward 5′-ACTCTTGGACTTTCAGGAAGG-3, Reverse 5′-TCTTTATAAGGGTCAATGTCCAT-3′. Primer core promoter region: Forward 5′-GGAAGGTCAATGACCTGGATC-3′, Reverse 5′- ATGCCTACAGCCTCCTAATAC-3′; Primer PreS1 promoter region: Forward 5′-CCCTATTATCCTGATAACGTGG-3′; Reverse 5′-GCTACGTGTGGATTCTCTCTT-3′. Primer X promoter region: Forward 5′-ATTGGCCATCAGCGCATGCG-3′; Reverse 5′-AGCTGCAAGGAGTTCCGCAGT-3′. Primer GAPDH promoter region: Forward 5′-GATGCCCCCATGTTTGTGAT-3′, Reverse 5′-GGTCATGAGCCCTTCCACAAT-3′.

### HBV DNA transient transfection

Full length HBV genotype F genome^25^ was released by SapI (Fermentas) digestion from pCR-XL-TOPO containing the HBV DNA. After digestion, the 3.2 kb DNA was purified with the Wizard SV Gel and PCR Clean-Up System (Promega) according to the manufacturer’s instructions. Huh-7 and HepG2 human hepatocarcinoma cells were seeded at a density of 6 × 10^5^ cells in 60 mm dishes and transfected after 24 h with 1.3 μg of digested HBV DNA using Lipofectamine 2000 (Life Technologies). Cells were harvested 24 h after transfection. For drug treatment, 0.5 mM pargyline hydrochloride was added to cells 16 h before and during HBV transfection. For shRNA treatment, 1.3 μg of pLL3.7 plasmid either with luciferase as control or SetDB1 target sequence (GCTCAGATGATAACTTCTGTA)^33^ was transfected with Lipofectamine 2000 48 h before HBV transfection. For siRNA treatment, 10 nM of either control siRNA (Silencer negative control #1 siRNA, Ambion, Life Technologies), human LSD1 siRNA (sc-60970, Santa Cruz Biotechnology), or human Set1A siRNA (sc-76484, Santa Cruz) was transfected with Lipofectamine.

For LSD1 and Set1A overexpression, cells were co-transfected with HBV DNA and either empty-, LSD1-FLAG, or FLAG-Set1A containing vector. The full-length HBV genotype F genome deficient in the expression of HBx was previously described^34^.

### Purification and analysis of HBV cytoplasmic intermediates and cccDNA

HBV intermediates were purified according to^35^. In brief, transfected cells were lysed with lysis buffer (10 mM Tris-Cl, pH 7.5; 50 mM NaCl; 1 mM EDTA; 0.5% Nonidet P40) in the presence of proteinase inhibitors for 4 min on ice. Nuclei were sedimented by centrifugation at 2,400 × g for 10 min. The supernatant was treated with 300 U DNase I (Winkler) for 1 h at 37 °C. Proteins were digested with 0.4 U of proteinase K (New England Biolabs) at 37 °C overnight and nucleic acids were purified by phenol–chloroform (1:1) extraction and ethanol precipitation. The nuclei were disintegrated vortexing them for 30 sec in nuclei lysis buffer (100 mM NaOH; 6% SDS), followed by incubation for 30 min at 37 °C. Sodium acetate was added to a final concentration of 600 mM and the pellet was discarded after centrifugation at 9,600 × g for 20 min. The cccDNA was purified twice by phenol-chloroform (1:1) extraction and then precipitated with ethanol. HBV intermediates were analyzed by real-time PCR (KAPA SYBR FAST, Universal qPCR kit).

### ELISA

Post-transfection supernatants were taken, and samples analyzed by ARCHITECT i1000 (Abbott) to detect either HBsAg or HBeAg, to obtain a signal-to-cutoff ratio, and then expressed as a % of untreated cells.

### cccDNA chromatin immunoprecipitation assays

Chromatin immunoprecipitation was performed according to^36^ with modifications. In brief, transfected cells were crosslinked for 10 min with 1% formaldehyde at room temperature. The reaction was quenched with glycine at a final concentration of 125 mM. For the analysis of LSD1 and Set1A recruitment, cells were then crosslinked with 2 mM ethylene glycol-bis-succinic acid N-hydroxyssuccinimide ester (Santa Cruz Biotechnology) at room temperature for 1 h. Then, the cells were washed twice with 1x PBS, resuspended in lysis buffer (5 mM Hepes, pH 8.0; 85 mM KCl; Triton X-100 and proteinase inhibitors) and homogenized with a Dounce homogenizer 10 times using a loose pestle. The cell extract was collected by centrifugation at 5,400 × g for 1 min at 4 °C, resuspended in nuclei buffer (50 mM Tris-HCl, pH 8.0; 10 mM EDTA; 1% SDS and protease inhibitors) and incubated for 10 min on ice. IP dilution buffer (20 mM Tris-HCl, pH 8.0; 2 mM EDTA; 50 mM NaCl; 1% Triton X-100; 0.1% SDS and protease inhibitors) was added and chromatin was sheared at high power for 4 pulses of 5 min in a Bioruptor water bath sonicator (Diagenode Inc., Denville, NJ, USA) to obtain fragments of 400 bp or smaller, and centrifuged twice at 16,000 × g for 10 min at 4 °C. Supernatant was collected and pre-cleared by incubating with 2 μg of IgG and 20 uL of protein A (Merck) for 2 h at 4 °C with rotation. The supernatant was immunoprecipitated with specific antibodies for 12–16 h at 4 °C. Immunocomplexes were recovered with the addition of 20 μL of protein A or G (for rabbit or mouse IgG, respectively) agarose beads and 1 h incubation with rotation at 4 °C. Immunoprecipitated complexes were washed once with sonication buffer (50 mM Hepes, pH 7.9; 140 mM NaCl; 1 mM EDTA, pH 8.0; 1% Triton X-100; 0.1% sodium deoxycholate; 1% SDS), twice with LiCl buffer (100 mM Tris-HCl pH 8.0; 500 mM LiCl; 1% Nonidet P40; 0.1% sodium deoxycholate) and once with TE buffer (50 mM Tris–HCl, pH 8.0; 2 mM EDTA). In case of reChIP, the Flag-HBx protein-DNA complexes were eluted by incubating with 0.2 mg/mL of 3xFlag-peptide (Sigma) followed by incubation with specific antibodies for 12–16 h at 4 °C, continuing the procedure as described. The protein-DNA complexes were eluted with elution buffer (50 mM NaHCO_3_; 1% SDS). NaCl was added to a final concentration of 200 mM. To reverse the crosslinking, immunoprecipitated complexes were incubated 12–16 h at 65 °C in the presence of 10 ug of RNase A (Invitrogen). Proteins were digested with 25 μg of proteinase K for 2 h at 50 °C. DNA was recovered with the Zymo Research Kit for DNA Clean & Concentrator and analyzed by real-time PCR (KAPA SYBR FAST, Universal qPCR kit). Data were processed as follows: from the Ct value obtained with the qPCR, we quantified viral DNA immunoprecipitated with respect to a standard curve prepared with the HBV genome. We then eliminated the background value obtained with the IgG control. We normalized the values with GAPDH as a loading control and with cccDNA. In the case of analyzing histone modifications, we normalized the data against the immunoprecipitated H3.

### Immunofluorescence

24 h after HBV transfection Huh-7 cells were fixed with 2% paraformaldehyde for 20 min and washed three times with 1x PBS. Cells were permeabilized with 0.5% Triton X-100 for 5 min, washed twice with 1x PBS and unspecific binding sites were blocked with 3% BSA. Cells were incubated overnight at 4 °C with antibody against hepatitis B virus core antigen (HBcAg), then rinsed three times with 1x PBS and incubated for 1 h with secondary antibody conjugated with Alexa 488 (Rockland). The cells were incubated with DAPI for 5 min and washed with PBS. Samples were imaged in an Olympus BX51 microscope and HBcAg immunofluorescence was quantified with Image J software.

## Additional Information

**How to cite this article**: Alarcón, V. *et al*. The enzymes LSD1 and Set1A cooperate with the viral protein HBx to establish an active hepatitis B viral chromatin state. *Sci. Rep.* **6**, 25901; doi: 10.1038/srep25901 (2016).

## Supplementary Material

Supplementary Information
