# Supplementary material for: The enzymes LSD1 and Set1A cooperate with the viral protein HBx to establish an active hepatitis B viral chromatin state
Source: Sci Rep. 2016 May 13;6:25901. doi: 10.1038/srep25901 (PMC4865824; doi:10.1038/srep25901)

The histone demethylase LSD1 and the viral protein HBx cooperate to establish an active hepatitis B viral chromatin state

Valentina Alarcón[1], Sergio Hernández[2], Lorena Rubio[1], Francisca Alvarez[1], Yvo Flores[1], Manuel Varas-Godoy[3], Giancarlo V. De Ferrari[4], Michael Kann[5,6,7], Rodrigo A. Villanueva[2*], and Alejandra Loyola[1,8*]

[1]Fundación Ciencia & Vida, 7780272, Santiago, Chile.

[2]Laboratorio de Virus Hepatitis, Departamento de Ciencias Biológicas, Facultad de Ciencias Biológicas, Universidad Andres Bello, Santiago, Chile.

[3]Centro de Investigación Biomédica, Facultad de Medicina, Universidad de los Andes, Santiago, Chile.

[4]Centro de Investigaciones Biomédicas. Facultad de Ciencias Biológicas y Facultad de Medicina. Universidad Andres Bello, Santiago, Chile.

[5]Univ. de Bordeaux, Microbiologie Fondamentale et Pathogénicité, UMR 5234, Bordeaux, France.

[6]CNRS, Microbiologie Fondamentale et Pathogénicité, UMR 5234, Bordeaux, France.

[7]CHU de Bordeaux, Bordeaux, France.

[8]Universidad San Sebastián, Santiago, Chile.

*To whom correspondence should be addressed.

Tel: (562) 27703408; Email: rodrigo.villanueva@unab.cl

Tel: (562) 23672048; Fax: (562) 22372259; Email: aloyola@cienciavida.org

Supplementary Figure 1. (A) Pargyline does not affect viability. Cellular viability after treatment was determined by trypan blue staining. The results are shown as % of living cells. The standard deviation was determined from three independent experiments. (B) Specificity of LSD1 DNA association. Human hepatocarcinoma Huh7 cells were transfected with a plasmid encoding a Flag-tagged LSD1. Chromatin Immunoprecipitation (ChIP) analysis was performed using Flag-beads or IgG-beads as a negative control. Immunoprecipitated DNA was quantified by qPCR using the commercially available human negative control primer (Active Motif, catalogue number #71001) and the human positive control primer set ACTB-2 (Active Motif, catalogue number #71001), as detailed in Material and Methods. The results are expressed as % of input. The standard deviation was obtained from three independent experiments.

Supplementary Figure 1

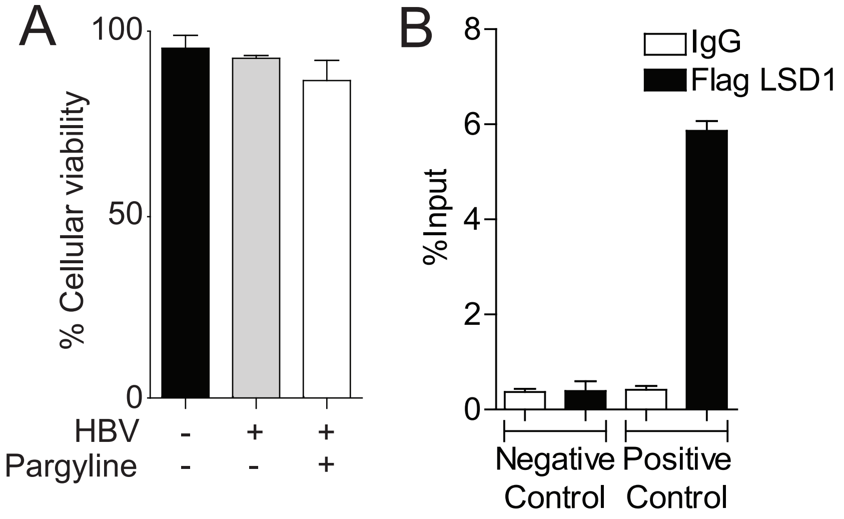

Supplement: Supplementary Information [file srep25901-s1.pdf]
